# Paroxetine Induces Apoptosis of Human Breast Cancer MCF-7 Cells through Ca^2+^-and p38 MAP Kinase-Dependent ROS Generation

**DOI:** 10.3390/cancers11010064

**Published:** 2019-01-09

**Authors:** Young-Woo Cho, Eun-Jin Kim, Marie Merci Nyiramana, Eui-Jung Shin, Hana Jin, Ji Hyeon Ryu, Kee Ryeon Kang, Gyeong-Won Lee, Hye Jung Kim, Jaehee Han, Dawon Kang

**Affiliations:** 1 Department of Physiology, College of Medicine and Institute of Health Sciences, Gyeongsang National University, Jinju 52727, Korea; ywcho@kbiohealth.kr (Y.-W.C.); eunjin1981@hanmail.net (E.-J.K.); mariemerci0222@gmail.com (M.M.N.); eui-jung@naver.com (E.-J.S.); wlgus9217@naver.com (J.H.R.); jheehan@gnu.ac.kr (J.H.); 2 Department of Convergence Medical Science, Gyeongsang National University, Jinju 52727, Korea; 3 Department of Pharmacology, College of Medicine and Institute of Health Sciences, Gyeongsang National University, Jinju 52727, Korea; hanajin.kr@daum.net (H.J.); hyejungkim@gnu.ac.kr (H.J.K.); 4 Department of Biochemistry, College of Medicine and Institute of Health Sciences, Gyeongsang National University, Jinju 52727, Korea; kkang@gnu.ac.kr; 5 Division of Hematology-Oncology, Department of Internal Medicine, College of Medicine and Institute of Health Sciences, Gyeongsang National University, Jinju 52727, Korea; brightree24@gmail.com

**Keywords:** apoptosis, breast cancer, calcium, depression, MAPK, paroxetine, reactive oxygen species

## Abstract

Depression is more common in women with breast cancer than the general population. Selective serotonin reuptake inhibitors (SSRIs), a group of antidepressants, are widely used for the treatment of patients with depression and a range of anxiety-related disorders. The association between the use of antidepressant medication and breast cancer is controversial. In this study, we investigated whether and how SSRIs induce the death of human breast cancer MCF-7 cells. Of the antidepressants tested in this study (amitriptyline, bupropion, fluoxetine, paroxetine, and tianeptine), paroxetine most reduced the viability of MCF-7 cells in a time-and dose-dependent manner. The exposure of MCF-7 cells to paroxetine resulted in mitochondrion-mediated apoptosis, which is assessed by increase in the number of cells with sub-G1 DNA content, caspase-8/9 activation, poly (ADP-ribose) polymerase cleavage, and Bax/Bcl-2 ratio and a reduction in the mitochondrial membrane potential. Paroxetine increased a generation of reactive oxygen species (ROS), intracellular Ca^2+^ levels, and p38 MAPK activation. The paroxetine-induced apoptotic events were reduced by ROS scavengers and p38 MAPK inhibitor, and the paroxetine’s effect was dependent on extracellular Ca^2+^ level. Paroxetine also showed a synergistic effect on cell death induced by chemotherapeutic drugs in MCF-7 and MDA-MB-231 cells. Our results showed that paroxetine induced apoptosis of human breast cancer MCF-7 cells through extracellular Ca^2+^-and p38 MAPK-dependent ROS generation. These results suggest that paroxetine may serve as an anticancer adjuvant to current cancer therapies for breast cancer patients with or without depression.

## 1. Introduction

Breast cancer is one of the most frequently occurring malignancies in women throughout the world [1]. As the incidence of breast cancer has increased steadily over the years, necessitating the development of various novel therapies. New potent anticancer drugs have been developed, but most of these drugs show cytotoxicity to normal cells as well as cancer cells. Development of anticancer drugs or adjuvants, which act more specifically on cancer cells, is really required to reduce the side-effects of anticancer drugs.

Breast cancer is highly associated with depression compared to other types of cancers, because breast cancer patients suffer psychological effects owing to changes in body shape [2]. Depression is more common in patients with cancer than the general population [3,4], and depression in breast cancer patients causes poor quality of life [5]. In addition, according to the American Cancer Society depression makes the person with cancer less able to follow their cancer treatment plan [6]. Therefore, mental health treatments are needed for cancer patients with depression.

Antidepressants have been used for the treatment of depression in cancer patients, but they have been shown a negative effect on compliance with the anticancer treatment [4,7]. Considering the interaction among antidepressants and anticancer drugs, the use of antidepressants with anticancer effects may reduce their side effects in the treatment of depressed people with cancer. Antidepressants have shown anticancer potential in a variety of cancer cells, but the use of antidepressants for the treatment of cancer is controversial among researchers. Some studies show that use of antidepressants increases breast cancer risk, but some studies do not agree with this view [8,9]. However, these studies have focused on cancer risk which could be seen when people experiencing depression take antidepressants.

Anticancer effects of antidepressants remain to be addressed in breast cancer patients with or without depression. If antidepressants have cytotoxic effect on breast cancer cells, the use of antidepressants for breast cancer patients regardless of depression is likely to give additive positive effect on treatment of breast cancer. This study was designed to investigate whether antidepressants induce cell death in breast cancer cells, and if so, to explore the potential mechanisms underlying antidepressant-induced cell death. In addition, the combinatorial effect of antidepressants and chemotherapeutic drugs was investigated.

## 2. Results

### 2.1. Apoptotic Death of Breast Cancer MCF-7 Cells Induced by Paroxetine Treatment

**Figure 1:**
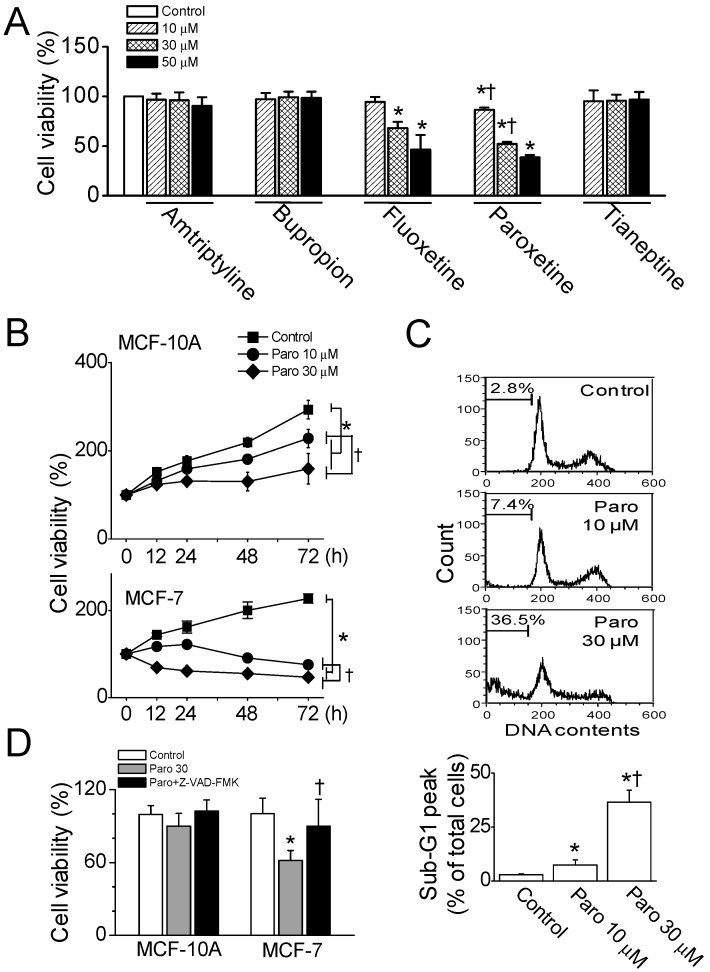
Paroxetine-induced death of MCF-7 cells. (**A**) Effect of antidepressants on cell viability of MCF-7 cells. Cell viability was analyzed using the MTT assay, as described in the Materials and Methods section. Cells were exposed to antidepressants (10, 30, or 50 μM) for 24 h. Cell viability was calculated as the percentage compared to the control. Each bar represents the mean ± SD of five independent experiments. * *p* < 0.05 compared to the control, which was not treated with antidepressants. ^†^ *p* < 0.05 compared between fluoxetine and paroxetine treatment at same concentration; (**B**) Time-dependent effect of paroxetine on normal and cancer cells. MCF-10A and MCF-7 cells were cultured in the presence or absence of paroxetine (10 or 30 μM) for 72 h. At the indicated time, cell viability was analyzed. The data represent the mean ± SD of five independent experiments; (**C**) Dose-dependent cytotoxic effect of paroxetine on MCF-7 cells. Sub-G1 content, which is considered as an indication of apoptosis, was analyzed using a FACSCalibur flow cytometer (upper panel) and quantified (lower panel). The cells were exposed to 10 or 30 μM paroxetine for 12 h; (**D**) Caspase-dependent cell death by paroxetine. Each bar represents the mean ± SD of three independent experiments. * *p* < 0.05 compared to each corresponding control. ^†^ *p* < 0.05 compared to paroxetine in MCF-7 cells. ‘Paro’ represents paroxetine.

To identify whether antidepressants induce the death of breast cancer cells, various antidepressants were screened using MCF-7 cells. The cells were cultured for 24 h in the presence of different concentrations of antidepressants (10 to 50 μM). When the cells were treated with 10, 30, or 50 μM antidepressant, cell death was visible following selective serotonin reuptake inhibitor (SSRI; fluoxetine and paroxetine) treatments. Fluoxetine and paroxetine treatments significantly decreased cell viability in a dose-dependent manner (*n* = 5, *p* < 0.05, Figure 1A). In comparison with fluoxetine’s effect, paroxetine induced significantly more cell death at all tested concentrations (10, 30, and 50 μM; *p* < 0.05). The treatment of MCF-7 cells with 10, 30, and 50 μM paroxetine resulted in cell viability of 86.5%, 52.1%, and 38.5%, respectively, compared to the control (Figure 1A). As shown in Figure 1A, amitriptyline and bupropion failed to induce cell death. Tianeptine, a selective serotonin reuptake enhancer that is used as an antagonist of SSRIs, did not induce cell death. Subsequent experiments focused on paroxetine’s effects.

The cytotoxicity of paroxetine shown in MCF-7 cells was evaluated in the normal mammary epithelial cell line MCF-10A. MCF-7 and MCF-10A cells were treated with paroxetine (10 or 30 μM) for 72 h. The treated MCF-7 cells and MCF-10A exhibited a significant decrease in cell viability at both concentration of paroxetine compared to control as time passed (*n* = 5, *p* < 0.05, Figure 1B). MCF-10A cells proliferated less in response to paroxetine treatment compared to control, whereas MCF-7 cells died, with paroxetine at 10 μM inducing cell death following exposure for over 24 h, while paroxetine at 30 μM induced cell death following 12 h exposure.

**Figure 2:**
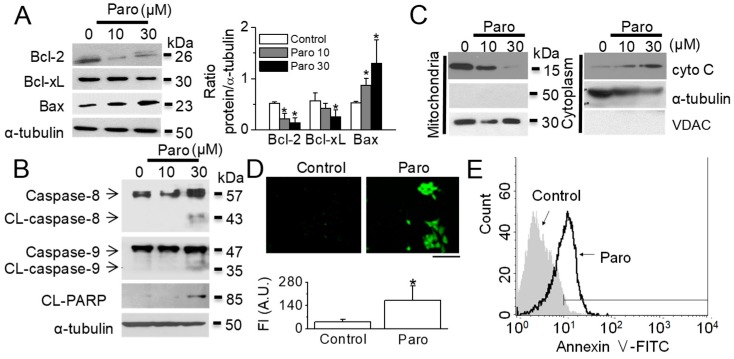
Paroxetine-induced apoptosis in MCF-7 cells. (**A**) Paroxetine-induced upregulation of proapoptotic proteins and downregulation of anti-apoptotic proteins. Cells were treated with paroxetine for 12 h. Western blot analyses were performed with the indicated antibodies (against Bcl-2, Bcl-xL, BAX, and α-tubulin) after treatment with different concentrations of paroxetine (10 or 30 μM); (**B**) Cleavage of caspases and PARP by paroxetine treatment. Cells were treated with paroxetine for 12 h. Western blot analyses were performed with the indicated antibodies (against caspase-8, caspase-9, cleaved PARP, and α-tubulin) after treatment with different concentrations of paroxetine (10 or 30 μM); (**C**) Release of cytochrome C (cyto C) from mitochondria into the cytoplasm by paroxetine treatment. To detect the expression of cyto C, cells were treated with paroxetine (10 or 30 μM) for 12 h and then fractionated into mitochondrial (Mito) and cytosolic (Cyto) components. Fractions were analyzed to assess the release of cyto C. An aliquot of total cell lysate (30 μg of protein per lane) was analyzed by immunoblotting; (**D**) Paroxetine-induced upregulation of caspase activity. Representative photomicrographs of MCF-7 cells labeled with FAM-VAD-FMK fluorescent dye after exposure of the cells to 30 μM paroxetine for 12 h. The scale bar indicates 50 µm. Each bar is the mean ± SD obtained from three independent experiments. * *p* < 0.05 compared to the control; (**E**) Paroxetine-induced apoptotic cell death. Cells were incubated with Annexin V-FITC and PI to evaluate early and late apoptosis. The percentage of apoptosis was quantified by the FACS analysis after 30 μM paroxetine treatment for 12 h. ‘Paro’ and ‘CL’ represent paroxetine and cleaved, respectively. ‘FI’ and ‘AU’ represent fluorescent intensity and arbitrary unit, respectively.

As shown in Figure 1C, paroxetine (10 or 30 μM) treatment for 12 h significantly increased the sub-G1 peak in MCF-7 cells (*n* = 3, *p* < 0.05). Treatment with paroxetine at concentrations of 10 and 30 μM yielded sub-G1 peaks of 7.4 ± 2.9% and 36.5 ± 3.6%, respectively. Caspase-dependent cell death was examined in the MCF-10A and MCF-7 cells pretreated with 20 μM Z-VAD-FMK, a cell-permeable pan caspase inhibitor, before paroxetine (30 μM) treatment for 12 h. In MCF-7 cells, paroxetine-induced cell death was significantly recovered by Z-VAD-FMK treatment (*n* = 6, *p* < 0.05, Figure 1D). Apoptotic signals related to paroxetine-induced cell death were analyzed using immunoblotting assay. Paroxetine treatment decreased the expression levels of Bcl-2 and Bcl-xL, anti-apoptotic proteins, and increased the expression level of pro-apoptotic Bax protein dose-dependently (Figure 2A). Caspases, a family of cysteine acid proteases, have been known to act as important mediators of apoptosis and to contribute to formation of the overall apoptotic morphology by cleavage of various cellular substrates. Because MCF-7 cells do not express caspase-3 [10], caspase-8 and caspase-9 were screened in MCF-7 cells. The exposure of MCF-7 cells to paroxetine at a concentration of 30 μM resulted in the cleavage of caspase-8 and caspase-9 (Figure 2B). Caspase activation induces poly (ADP-ribose) polymerase (PARP) cleavage, which serves as a biochemical marker of cells undergoing apoptosis. As shown in Figure 2B, paroxetine treatment increased the 85 kDa fragment of PARP resulting from caspase cleavage.

Cells treated with paroxetine were fractionated into cytosolic and mitochondrial fractions to detect the translocation of cytochrome C during apoptosis. Paroxetine treatment showed that cytochrome C expression decreased in mitochondrial fraction and concomitantly accumulated in the cytosolic fraction. Mitochondrial fraction was confirmed by no expression of tubulin and expression of voltage-dependent anion channel (VDAC), which is expressed in the outer mitochondrial membrane (Figure 2C). To visualize paroxetine-induced apoptotic cells, caspase activity was measured using a FLICA apoptosis kit (Immunochemistry Technologies; Bloomington, MN, USA). Paroxetine treatment (30 μM) significantly increased caspase activity compared to the control (*n* = 30, Figure 2D). The percentage of early and late apoptotic cells was increased by 18.6 ± 9.2-fold after treatment with 30 μM paroxetine (*n* = 3, Figure 2E). Early apoptotic cells showed Annexin V-FITC^+^/PI^−^ staining pattern (60.0 ± 10.6%), and late apoptotic cells displayed Annexin V-FITC^+^/PI^+^ staining (40.0 ± 10.6%).

### 2.2. Paroxetine-Induced ROS Generation and Increase in Intracellular Ca^2+^ Levels

**Figure 3:**
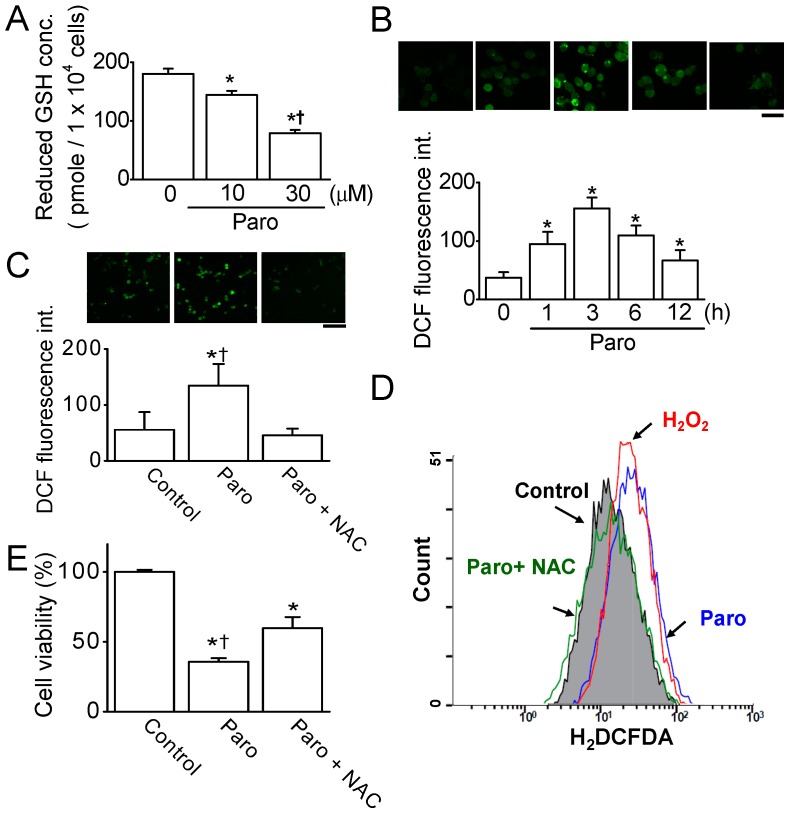
Paroxetine-induced ROS generation in MCF-7 cells. (**A**) The reduced glutathione concentration was dose-dependently reduced by paroxetine treatment in MCF-7 cells. The reduced glutathione concentration was measured in cells treated with 10 or 30 μM paroxetine using a total glutathione concentration analysis kit. The calculation of the reduced glutathione concentration is described in the Materials and Methods section; (**B**) Paroxetine-induced ROS generation was maintained for a long time. Cells were treated with 30 μM paroxetine for 12 h. ROS generation was measured at the indicated time. The scale bar indicates 50 μm; (**C**) Paroxetine-induced ROS generation was inhibited by NAC pretreatment. Cells were incubated with H_2_DCFDA to evaluate ROS generation. The ROS levels in the cells were quantified by the measurement of fluorescent intensity using fluorescence microscopy (**C**) and FACS analysis (**D**) after 30 μM paroxetine treatment for 3 h (positive control; H_2_O_2_ at 100 μM for 3 h); (**E**) Reduction in paroxetine-induced cell death by NAC treatment. NAC (3 mM) pretreatment was performed for 2 h before paroxetine (30 μM) treatment. The cells were exposed to paroxetine for 12 h. ‘Paro’ represents paroxetine. * *p* < 0.05 compared to the control; ^†^ *p* < 0.05 compared to the paroxetine plus NAC treatment.

ROS are involved in the modulation of apoptosis [11], and glutathione plays a role in controlling ROS generation [12]. To investigate whether paroxetine generates ROS in MCF-7 cells, cells were monitored using the oxidation-sensitive fluorescent dye H_2_DCFDA after paroxetine treatment. As shown in Figure 3A, reduced glutathione concentration was significantly decreased in the cells exposed to 10 or 30 μM paroxetine compared to the control (*n* = 5, *p* < 0.05). During paroxetine treatment for 1, 3, 6, and 12 h, ROS generation was high compared to ROS levels in the control, and the highest ROS generation was observed after 3 h of paroxetine treatment (*n* = 5, *p* < 0.05, Figure 3B). Subsequent experiments measuring ROS were performed at 3 h of paroxetine treatment. The paroxetine-induced ROS generation was significantly decreased by 64.0 ± 12.4% in the pretreatment with *N*-acetyl-l-cysteine (NAC), a ROS scavenger (*n* = 5, *p* < 0.05, Figure 3C). FACS analysis also showed that paroxetine (30 μM) induced a similar amount of ROS generation as did H_2_O_2_ (100 μM), which was used as a positive control, and NAC pretreatment decreased the paroxetine-induced ROS generation (Figure 3D). 

To identify whether ROS generation is associated with paroxetine-induced apoptosis in MCF-7 cells, cells were cultured in the presence or absence of NAC. Paroxetine-induced cell death was significantly reduced by NAC pretreatment (*n* = 5, *p* < 0.05, Figure 3E). Numerous studies have shown that Ca^2+^ is essential for ROS generation [13]. 

**Figure 4:**
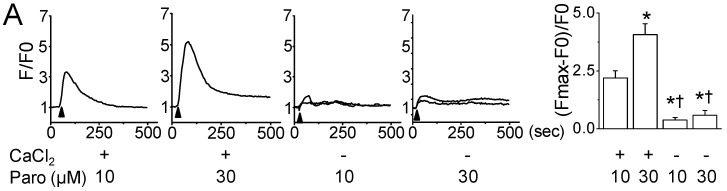

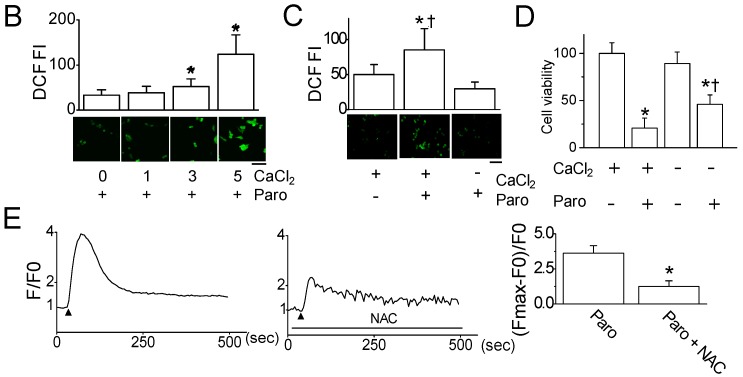
Paroxetine-induced ROS generation affected by extracellular Ca^2+^ concentration. (**A**) Paroxetine-induced intracellular Ca^2+^ increase in MCF-7 cells. Cells were loaded with fluo-3 AM for 45 min to evaluate changes in Ca^2+^ levels. Paroxetine (10 or 30 μM) was applied to the bath solution in the presence or absence of CaCl_2_ (1 mM). EGTA (1 mM) was used to remove extracellular free Ca^2+^ to form a Ca^2+^-free condition. The bar graphs show the changes in [Ca^2+^]_i_ displayed as fluorescence intensity (F) using a Fluoview software program. The calculation method is described in the Materials and Methods section. * *p* < 0.05 compared to the CaCl_2_ + 10 μM paroxetine. ^†^ *p* < 0.05 compared to the each corresponding control (CaCl_2_ + same concentration of paroxetine); (**B**) Effect of extracellular Ca^2+^ concentration on paroxetine-induced ROS generation. MCF-7 cells were stained with H_2_DCFDA to evaluate ROS generation. Paroxetine (30 μM) was added to the cells in the presence or absence of the indicated CaCl_2_ concentration. The ROS levels in each cell were quantified by fluorescence microscopy after 3 h of paroxetine treatment. The 1, 3, or 5 mM CaCl_2_ was added to a homemade physiological solution with the following (in mM): NaCl (125), KCl (3), MgCl_2_ (1), HEPES (10), and glucose (5). The osmolarity and pH of the solution were adjusted to 280 mOsm and 7.3, respectively. * *p* < 0.05 compared to the 0 CaCl_2_ + 30 μM paroxetine; (**C**) Suppression of paroxetine-induced ROS generation under extracellular Ca^2+^ free condition. EGTA (1 mM) was used to remove extracellular free Ca^2+^. * *p* < 0.05 compared to the CaCl_2_ + 0 μM paroxetine. ^†^ *p* < 0.05 compared to the CaCl_2_ + 30 μM paroxetine; (**D**) Effect of extracellular Ca^2+^ concentration on paroxetine-induced cell death. The cells were cultured in DMEM with and without CaCl_2_ to rule out the effect of nutrients on cell death for 12 h. * *p* < 0.05 compared to the each corresponding control. ^†^ *p* < 0.05 compared to the CaCl_2_ + paroxetine; (**E**) Inhibition of paroxetine-induced Ca^2+^ influx by NAC treatment. Cells were incubated with fluo-3 AM to evaluate changes in Ca^2+^ levels. Paroxetine (30 μM) was added to the bath medium (RPMI) in the presence or absence of 3 mM NAC. Ca^2+^ influx into the cells was quantified by the Fluoview software program. The arrowheads indicate the addition of paroxetine. The plus and minus signs (+ and −) represent treatment conditions with and without CaCl_2_, respectively. ‘Paro’ and ‘FI’ represents paroxetine and fluorescence intensity, respectively. * *p* < 0.05 compared to the paroxetine. Each bar is the mean ± SD obtained from three independent experiments.

To investigate whether paroxetine-induced ROS generation is related to changes in intracellular Ca^2+^ concentration ([Ca^2+^]_i_), levels of [Ca^2+^]_i_ and ROS in MCF-7 cells were measured using the calcium indicator fluo 3-AM and H_2_DCFDA, respectively, before and after paroxetine treatment. As shown in Figure 4A, paroxetine treatment increased intracellular Ca^2+^ levels. A high concentration of paroxetine (30 μM) produced a higher transient Ca^2+^ peak than a low concentration of paroxetine (10 μM) did. The paroxetine-induced increase in intracellular Ca^2+^ level was significantly reduced in the external Ca^2+^ free condition, which is produced by adding EGTA (1 mM), a specific chelator for Ca^2+^, to bath solution without CaCl_2_ (*n* = 30, *p* < 0.05), indicating that paroxetine-induced Ca^2+^ increase mainly results from Ca^2+^ influx through the plasma membrane. To identify the relationship between ROS generation and [Ca^2+^]_i_, the DCF fluorescence intensity was measured in the presence of different extracellular Ca^2+^ concentration ([Ca^2+^]_e_). The cells were exposed to paroxetine in the presence of 0, 1, 3, or 5 mM CaCl_2_. As shown in Figure 4B, paroxetine treatment enhanced ROS generation as [Ca^2+^]_e_ increased. With the addition of 3 or 5 mM CaCl_2_, ROS generation significantly increased by 1.6-and 3.7-fold, respectively, compared to the control, which contained free Ca^2+^ in the physiological solution (*n* = 18, *p* < 0.05). In the Ca^2+^ free condition, paroxetine failed to generate ROS (*n* = 18, Figure 4C). Paroxetine induced lower cell death in the low [Ca^2+^]_e_, which is 0 mM CaCl_2_ culture medium not containing EGTA, compared to normal culture media containing ~2 mM CaCl_2_. The paroxetine-induced cell death was decreased by 55 ± 6% at 12 h incubation in the low [Ca^2+^]_e_, indicating that paroxetine-induced cell death is dependent on [Ca^2+^]_e_ (*n* = 4, Figure 4D). The relationship between ROS generation and [Ca^2+^]_i_ was conversely examined. In the presence of NAC, a ROS scavenger, paroxetine induced a limited increase in intracellular Ca^2+^ levels compared to a condition without NAC (3.63 ± 0.51 in the absence of NAC versus 1.24 ± 0.41 in the presence of NAC, *n* = 30, Figure 4E).

### 2.3. Paroxetine-Induced Mitochondrial Dysfunction in MCF-7 Cells

**Figure 5:**
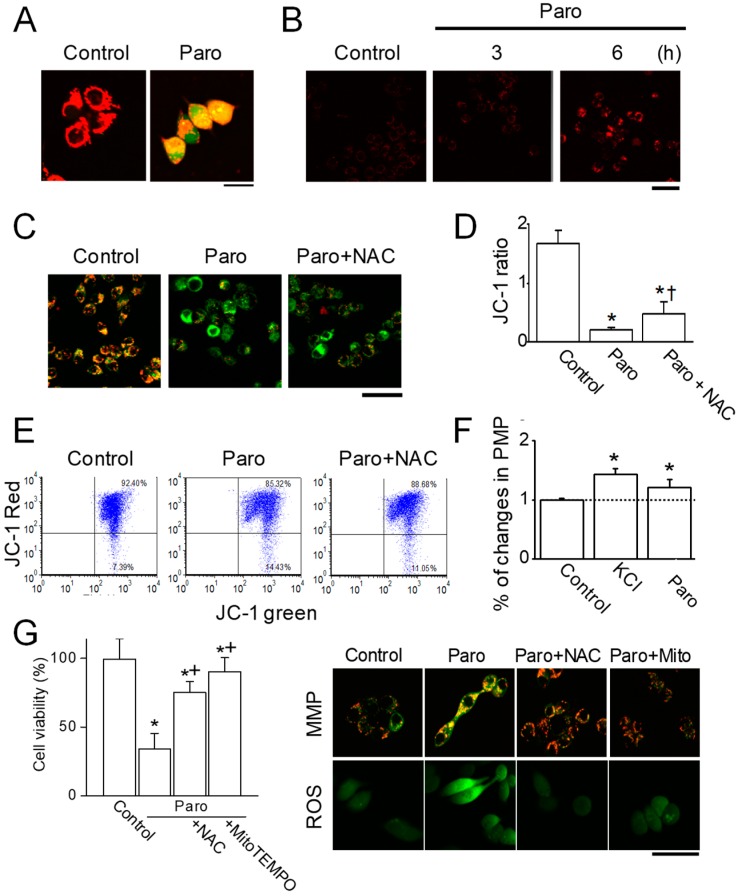
Paroxetine-induced mitochondrial ROS generation and MMP depolarization. (**A**) Mitochondria were a source of ROS generation by paroxetine treatment. The cells were exposed to paroxetine (30 μM) for 12 h. The cells were incubated with MitoTracker Red dye and H_2_DCFDA for 30 min in the presence or absence of paroxetine (30 μM). Red, green, and yellow indicate mitochondrial regions, ROS generation, and mitochondrial regions showing ROS generation (merged color), respectively. The scale bar indicates 20 μm; (**B**) The mitochondrial ROS generation was confirmed using specific mitochondrial ROS dye, MitoSOX. Cells were incubated with MitoSOX after paroxetine (30 μM) treatment for indicated time periods. The scale bar represents 50 μm. (**C**,**D**) MCF-7 cells were incubated with a specific MMP dye, JC-1, in the presence or absence of paroxetine and/or NAC. The cells were exposed to paroxetine and/or NAC for 12 h. NAC pretreatment was performed before 2 h of paroxetine treatment. Green and red indicate JC-1 monomers and JC-1 aggregates, respectively. Changes in the MMP of the cells by paroxetine were analyzed using fluorescence microscopy (**C**) and quantified (**D**). The scale bar represents 100 μm; (**E**) Changes in the MMP of the cells by paroxetine were also analyzed using a FACSCalibur flow cytometer. The dot plots showed red fluorescence (JC-1 red aggregates) versus green fluorescence (JC-1 green monomer); (**F**) Changes in the plasma membrane potential (PMP) of cells by paroxetine treatment were measured with DiO membrane labeling dye. To induce depolarization of the membrane potential, KCl (25 mM) was used and compared to paroxetine’s effect (30 μM). The PMP of the cells was quantified by the Fluoview software program; (**G**) Effect of MitoTEMPO on paroxetine-induced cell death, ROS generation, and decrease in MMP. The scale bar represents 50 μm. ‘Paro’ and ‘Mito’ represent paroxetine and MitoTEMPO, respectively. Each bar is the mean ± SD obtained from three independent experiments. * *p* < 0.05 compared to the control; ^†^ *p* < 0.05 compared to the paroxetine.

To identify the intracellular sources of ROS generation due to paroxetine treatment, MCF-7 cells treated with 30 μM paroxetine were concomitantly stained with the ROS indicator H_2_DCFDA and MitoTracker, a mitochondrion-selective probe. ROS were generated in mitochondria in paroxetine-treated MCF-7 cells, as evaluated by the colocalization of H_2_DCFDA (green) and MitoTracker (red). Yellow and green showed ROS generations from mitochondrial regions and another cellular compartment such as cytoplasm, respectively, in response to paroxetine treatment (Figure 5A). The use of MitoSOX Red reagent, a highly selective mitochondrial ROS detection dye, also confirmed that the intracellular localization of ROS generation in paroxetine-treated MCF-7 cells was mitochondrial (Figure 5B). As shown in Figure 5B, significantly visible fluorescence signals were observed in the cells after 6 h of paroxetine treatment compared to untreated cells.

Increases in ROS generation can induce mitochondrial dysfunction, which is highly associated with a decline in the mitochondrial membrane potential (MMP) [14]. The MMP was measured using JC-1 cationic dye. As shown in Figure 5C, MCF-7 cells showed red and green fluorescence. Paroxetine treatment markedly changed the fluorescence color in cells from red to green, indicating depolarization of the MMP. Paroxetine treatment significantly reduced the JC-1 ratio, which represents the fluorescence intensity ratio (red/green), compared to the control treatment, whereas NAC pretreatment decreased the paroxetine-induced reduction in the JC-1 ratio (*p* < 0.05, *n* = 30, Figure 5C,D). To confirm the image analysis of the depolarization of the MMP by paroxetine, FACS analysis was performed. Figure 5E shows concomitant JC-1 red aggregates and JC-1 green monomers. The JC-1 green monomers were present in 7.4%, 14.4%, and 11.1% of cells for the control, paroxetine treatment, and the combination of paroxetine and NAC, respectively. The effect of paroxetine on the plasma membrane potential was also examined with DiO membrane labeling dye. A high concentration of KCl (25 mM) was used as a positive control for depolarization. Paroxetine treatment induced depolarization of the plasma membrane potential, like 25 mM KCl did (*n* = 15, Figure 5F). MitoTEMPO, a mitochondria specific ROS scavenger, reduced paroxetine-induced cell death, decrease in MMP, and ROS generation like NAC did (*n* = 3, Figure 5G).

### 2.4. Paroxetine-Induced ROS Generation Is Due to the Activation of p38 MAPK

In ROS generation, MAPKs are important cascades that facilitate signal transduction in cells [15,16]. To explore the underlying signal transduction in paroxetine-induced apoptosis in MCF-7 cells, the activation of MAPK (ERK1/2, JNK, and p38) in response to paroxetine treatment (30 μM) was measured by immunoblot analysis. 

**Figure 6:**
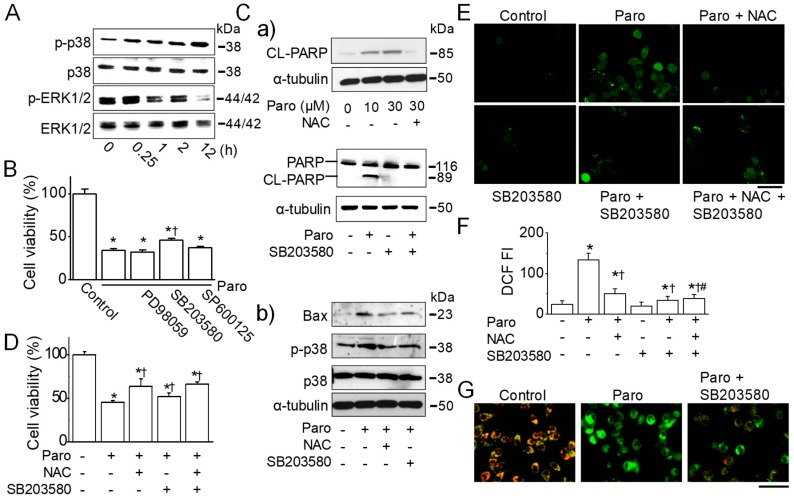
Paroxetine-induced apoptosis and ROS generation are mediated by p38 activation. (**A**) Effect of paroxetine on the phosphorylation of MAPK. MCF-7 was treated with 30 μM paroxetine for the indicated time periods. Activated p38 MAPK (p-p38) and ERK (p-ERK) were detected in immunoblots using antibodies specific for the phosphorylated form of each kinase. The same blot was stripped and used to determine the amount of each kinase; (**B**) Effect of MAPK inhibitors on paroxetine-induced cell death. Cells were pretreated with 10 μM SB203580, 10 μM PD98059, or 10 μM SP600125 for 30 min before 12 h treatment with 30 μM paroxetine. Cell viability was determined by an MTT assay. (**Ca**,**Cb**) Effect of NAC and SB203580 on PARP cleavage (**Ca**) and p38 activation (**Cb**). Protein extracts were prepared after treatment with or without 10 μM SB203580 and 3 mM NAC for 30 min, followed by treatment with or without 10 μM or 30 μM paroxetine for 12 h. Immunoblotting assays were performed with antibodies against PARP, cleaved PARP, Bax, p-p38, p38, and α-tubulin; (**D**) Effect of SB203580 and NAC on paroxetine-induced cell death. Cells were pretreated with 10 μM SB203580 and 3 mM NAC for 30 min before 12 h treatment with 30 μM paroxetine. Cell viability was determined by an MTT assay; (**E**,**F**) Reduction of ROS in MCF-7 cells by pretreatment with NAC, SB203580, or NAC and SB203580. MCF-7 cells were incubated with the ROS indicator H_2_DCFDA and then pretreated with 10 μM SB203580 and/or NAC for 30 min before 3 h treatment with 30 μM paroxetine. The changes in ROS levels in the cells were analyzed using fluorescence microscopy (**E**) and quantified (**F**); (**G**) Reduction in MMP depolarization in MCF-7 cells by SB203580. Cells were incubated with a specific MMP dye, JC-1, in the presence or absence of paroxetine and/or SB203580. The cells were exposed to paroxetine and/or SB203580 for 3 h. SB203580 pretreatment was performed before 30 min of paroxetine treatment. Green and red indicate JC-1 monomers and JC-1 aggregates, respectively. Changes in the MMP of the cells by paroxetine were analyzed using fluorescence microscopy. The scale bar represents 50 μm. ‘Paro’ represents paroxetine. * *p* < 0.05 compared to the control (no treatment); ^†^ *p* < 0.05 compared to the paroxetine. ^#^ *p* < 0.05 compared to paroxetine plus NAC. Each bar is the mean ± SD obtained from three independent experiments.

As shown in Figure 6A, the activation of p38 and ERK1/2 in response to paroxetine treatment was markedly increased at late time point (12 h) and an early time point (15 min), respectively. JNK was not activated by paroxetine treatment (data not shown). To evaluate whether p38 and ERK activations are associated with paroxetine-induced apoptosis, MCF-7 cells were pretreated with specific inhibitors of MAPK. Paroxetine-induced cell death significantly decreased by 12% in the presence of the selective p38 inhibitor SB203580 (65.7 ± 1.7% for paroxetine vs. 54.0 ± 1.9% for paroxetine+SB203580; *p* < 0.05, *n* = 6, Figure 6B). However, inhibitors of ERK (PD98059) and JNK (SP600125) had no effect on recovery of paroxetine-induced cell death.

Considering the inhibitory effect of NAC on paroxetine-induced apoptosis, the effect of p38 inhibitor was compared to that of NAC. NAC or SB203580 treatment reduced paroxetine-induced PARP cleavage (Figure 6Ca). Pretreatment with NAC offset the level of paroxetine-induced p38 activation to the control level, as SB203580 did (Figure 6Cb). Both NAC and SB203580 reduced paroxetine-induced BAX upregulation (Figure 6Cb). To identify the physiological role of p-p38 suppression, an MTT assay was performed. Pretreatment with SB203580 significantly reduced paroxetine-induced cell death (*p* < 0.05), but the effect was very small. As shown in previous experiments, NAC pretreatment also reduced paroxetine-induced cell death. The NAC effect was greater than the SB203580 effect. A combination of NAC and SB203580 slightly increased cell viability compared to chemical treatment alone (*n* = 6, Figure 6D).

To evaluate the role of p38 phosphorylation in the paroxetine-induced ROS generation and changes in the MMP, fluorescence image analysis was performed with H_2_DCFDA and JC-1 dye. Pretreatment with NAC (3 mM) and/or SB203580 (10 μM) significantly reduced paroxetine-induced ROS generation: approximately 66.2 ± 10.0% for NAC, 74.7 ± 7.1% for SB203580, and 71.0 ± 10.6% for NAC plus SB203580 (*p* < 0.05, *n* = 18, Figure 6E,F). Regarding the MMP, pretreatment with SB203580 reduced paroxetine-induced MMP depolarization (Figure 6G).

### 2.5. Synergistic Effect of Paroxetine on the MCF-7 Cell Death Induced by Anticancer Drugs

**Figure 7:**
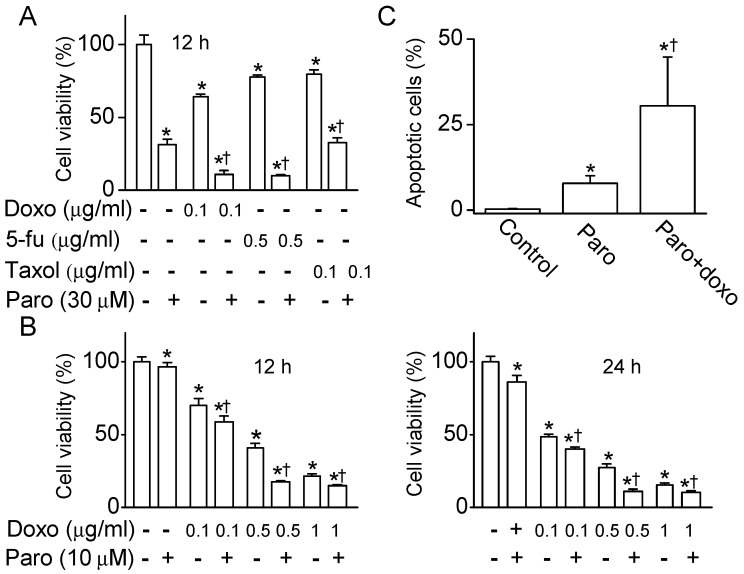
Synergistic effect of paroxetine on cell death induction by anticancer drugs. (**A**) Effect of anticancer drugs on the death of MCF-7 cells. MCF-7 ells were treated with an anticancer drug (doxorubicin (doxo, 0.1 μg/mL), 5-fluorouracil (5-Fu, 0.5 μg/mL), or taxol (0.1 μg/mL)) and/or paroxetine (30 μM) for 12 h. Cell viability was determined by an MTT assay; (**B**) Cell death of MCF-7 cells induced by paroxetine and doxorubicin treatment. Cells were treated with the indicated concentrations of doxo and/or paro for 12 h or 24h. * *p* < 0.05 compared to each corresponding control untreated with drugs. ^†^ *p* < 0.05 compared to group treated with only anticancer drug of same concentration; (**C**) High increase in apoptotic cell death in the combination of paroxetine and doxorubicin. The apoptotic cells were quantified by the FACS analysis with Annexin V-FITC and PI after 30 μM paroxetine treatment for 12 h. * *p* < 0.05 compared to the control. ^†^ *p* < 0.05 compared to the paroxetine treatment. Each bar is the mean ± SD obtained from three independent experiments. ‘Paro’ represents paroxetine.

To evaluate the potency of paroxetine for adjuvant chemotherapy, paroxetine and an anticancer drug for breast cancer (0.1 μg/mL doxorubicin, 0.5 μg/mL 5-fluorouracil, or 0.1 μg/mL taxol) were concomitantly treated to the cells. Treatment with paroxetine (30 μM) for 12 h induced more cell death than did anticancer drugs (Figure 7A). Combined treatment with paroxetine and an anticancer drug increased cell death compared to treatment with paroxetine or an anticancer drug alone (Figure 7A). Among the anticancer drugs tested in this study, doxorubicin was the most effective inducer of MCF-7 cell death. To assess the function of paroxetine as an adjuvant for chemotherapy, an MTT assay was performed at a lower concentration of paroxetine (10 μM), and cell viability was compared after 12 h and 24 h of combined treatment with doxorubicin and paroxetine. Doxorubicin treatment reduced cell viability in a dose-dependent manner. In addition, combination treatment with doxorubicin and paroxetine (10 μM) for 12 h reduced cell viability more than did doxorubicin alone. The effect was greater following treatment with doxorubicin and/or paroxetine for 24 h than for 12 h. Paroxetine showed a synergistic effect on the reduction in MCF-7 cell viability by anticancer drugs for both the 12 h and the 24 h treatments (Figure 7B). Annexin V-FITC and PI staining showed increase in percentage of early and late apoptotic cell death in paroxetine treatment, and the apoptotic cell death more increased in the combination of paroxetine and doxorubicin (Figure 7C). Necrotic cell death was also seen in the combination of paroxetine and doxorubicin. 

### 2.6. Apoptotic Death of Breast Cancer MDA-MB-231 Cells Induced by Paroxetine Treatment

**Figure 8:**
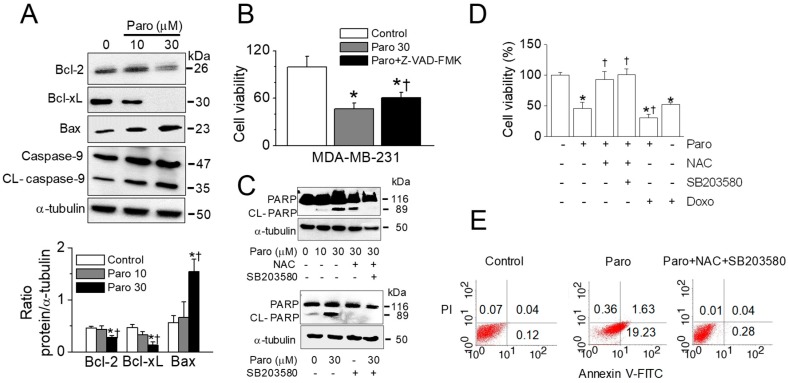
Paroxetine-induced apoptosis in MDA-MB-231 cells. (**A**) Paroxetine-induced downregulation of anti-apoptotic proteins and upregulation of proapoptotic protein. Bar graphs show the ratio of Bcl-2, Bcl-xL, and Bax against α-tubulin; (**B**) Effect of a pan caspase inhibitor on paroxetine-induced cell death; (**C**) Paroxetine-induced PARP cleavage; (**D**) Paroxetine-induced cell death reduced by treatment with a ROS scavenger and a p38 inhibitor; (**E**) Paroxetine-induced apoptotic cell death. The percentage of Annexin V-FITC positive cells was increased in the paroxetine treatment, but the percentage was decreased in the combination of NAC and SB203580. The numbers in the dot plots represent the % of early/late apoptosis and necrosis. Cells were treated with paroxetine for 12 h. A western blot analysis was performed with the indicated antibodies (Bcl-2, Bcl-xL, BAX, caspase-9, PARP, and α-tubulin) after treatment with different concentrations of paroxetine (10 or 30 μM). An aliquot of total cell lysate (30 μg of protein per lane) was analyzed by immunoblotting. Each bar is the mean ± SD obtained from three independent experiments. * *p* < 0.05 compared to the control. ^†^ *p* < 0.05 compared to the paroxetine 10 μM (**A**) or 30 μM (**B**,**D**).

To confirm the paroxetine-induced apoptotic death in breast cancer cells, another breast cancer cell line MDA-MB-231 was adopted. Paroxetine (30 μM) treatment significantly decreased the expression levels of Bcl-2 and Bcl-xL and increased the expression level of Bax protein (*n* = 3, *p* < 0.05). Caspase-9 was cleaved by paroxetine treatment dose-dependently (Figure 8A). Pretreatment with 20 μM Z-VAD-FMK significantly reduced paroxetine-induced cell death (*n* = 6, *p* < 0.05, Figure 8B). Paroxetine-induced PARP cleavage was shown in Figure 8C. NAC or/and SB203580 markedly reduced the PARP cleavage (Figure 8C). Paroxetine-induced cell death was reduced by NAC and SB203580 treatment, like NAC and SB203580 did in MCF-7 cells. The combination of paroxetine (30 μM) and doxorubicin (0.5 μg/mL) showed higher cell death than paroxetine- or doxorubicin single treatment (*n* = 3, *p* < 0.05, Figure 8D). Annexin V-FITC and PI staining showed that paroxetine treatment increased apoptotic cell death, and the combination of NAC and SB203580 reduced the paroxetine-induced apoptotic cell death (Figure 8E).

## 3. Discussion

**Figure 9:**
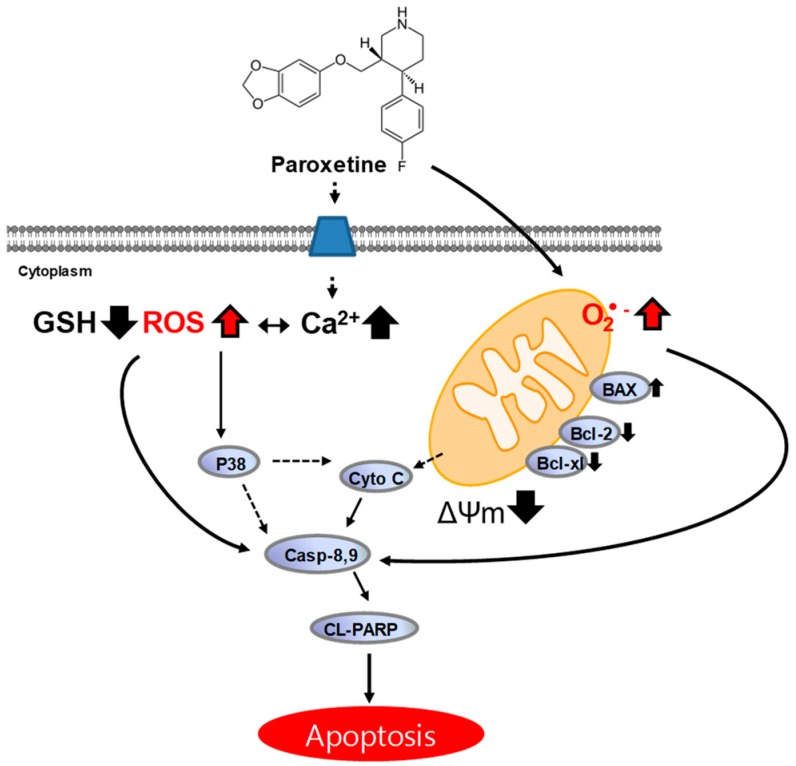
Schematic representation of paroxetine-induced apoptosis. Paroxetine induces intracellular and mitochondrial ROS generation and an increase in intracellular Ca^2+^ levels. The increase in ROS generation induces p38 MAPK activation and begins the apoptotic process by increasing the Bax/Bcl-2 ratio, causing the release of cytochrome c from mitochondria, and finally activating caspases and PARP cleavage.

This study demonstrates that paroxetine has anticancer potential in human breast cancer MCF-7 cells through ROS generation. In addition to ROS generation, an intracellular Ca^2+^ increase through Ca^2+^ influx and p38 MAPK activation are involved in paroxetine-induced apoptosis (Figure 9). The paroxetine-induced cell death is Ca^2+^-dependent event. Moreover, paroxetine gives a synergistic effect on cell death induced by chemotherapeutic drugs with a marked cytotoxic effect on breast cancer cells.

### 3.1. Cytotoxic Effect of Paroxetine on Breast Cancer Cells

Among the antidepressants tested in this study, the SSRIs fluoxetine and paroxetine showed anticancer effects. In particular, paroxetine showed a marked cytotoxic effect on breast cancer MCF-7 cells. Many types of SSRIs have been shown to have anticancer potential in various cancer cells [17,18,19,20,21], but reports on the anticancer effect of paroxetine in comparison with other SSRIs are rare. In particular, little is known regarding paroxetine’s effect at the cellular level in breast cancer. Some studies have shown that paroxetine induces cell death in human colorectal cancer cell lines [18,21], rat glioma and human neuroblastoma cells [22], and human hepatocellular carcinoma cells [20]. Paroxetine evokes cell death via apoptosis in human osteosarcoma cells by activation of the p38 MAPK and caspase-3 pathways, without the involvement of [Ca^2+^]_i_ elevation [23]. In another study, in Madin-Darby canine kidney cells, paroxetine caused protein kinase C-dependent, Ca^2+^-independent apoptosis, which was potentiated by inhibition of the ERK pathway [24]. Paroxetine was also found to induce death of OC2 human oral cancer cells in a Ca^2+^-independent manner [25]. In most studies, paroxetine increased [Ca^2+^]_i_, but paroxetine-induced cell death was not reversed when cytosolic and/or extracellular Ca^2+^ was chelated. The authors therefore insisted that paroxetine-induced cell death is a Ca^2+^-independent event. In agreement with earlier studies, our study also showed that paroxetine increased [Ca^2+^]_i_ and activation of p38 MAPK and caspases. However, paroxetine induced lower apoptotic cell death in the absence of extracellular CaCl_2_ than in the presence of extracellular CaCl_2_. The effect of paroxetine was compared between MCF-7 cells cultured in the DMEM media with and without CaCl_2_ which contain same concentration of amino acids, vitamins, inorganic salts, and D-glucose except for the CaCl_2_. These results indicate that paroxetine-induced cell death is Ca^2+^-dependent event. Similar to our findings, paroxetine induces apoptosis of rat astrocytes through increase in Ca^2+^ influx, decrease in MMP, and mitochondrial ROS production [26]. Many ROS-generating and antioxidant systems in living cells are dependent on [Ca^2+^]_i_ [27,28,29,30,31]. We previously reported at the AACR 107th Annual Meeting a study showing that paroxetine induced apoptotic death of MCF-7 cells through mitochondrial dysfunction and modulation of K^+^ channels without clear signaling pathways [32]. The current study is updated with clear apoptotic signaling pathways and synergistic effect of paroxetine on doxorubicin-induced cell death in MCF-7 and MDA-MB-231 cells. Both MCF-7 and MDA-MB-231 cells showed caspase-dependent cell death pathway, but caspase-independent pathway might be also present in paroxetine-induced cell death of MDA-MB-231 cells based on pan caspase inhibitor did not completely block the paroxetine-induced cell death (see Figure 8).

ROS, which are not only produced by various cellular events, but also derived from exogenous sources, play an important role in the regulation of cellular functions, such as cell proliferation, differentiation, and immune responses [28]. However, excessive generation of ROS causes oxidative stress, which contributes to adverse events including neuronal cell death [33,34]. Accumulating evidences suggest that the increase in oxidative stress is related to the apoptotic response induced by several anticancer agents. In addition, chemotherapeutic drugs are selectively toxic to cancer cells because human cancer cells appear to generate ROS at a greater rate than normal cells do in response to chemotherapeutic drugs [35,36]. The current study showed that paroxetine elevated the levels of intracellular ROS from mitochondria and other microcellular organisms that are dependent on [Ca^2+^]_e_. Paroxetine induced mitochondrion-mediated apoptosis, which is characterized by the cleavage of caspase-8 and caspase-9, an induction of PARP cleavage, a reduction in the MMP, and an increase in the Bax/Bcl-2 ratio through the induction of mitochondrial ROS generation. In addition, our results suggest that Ca^2+^ and ROS might be cross-talking messengers in the paroxetine-induced apoptosis in MCF-7 cells.

MAPKs have been associated with the regulation of cell fate though apoptosis [37]. These signaling pathways are activated by various stimuli, such as cytokines, UV irradiation, and anticancer agents. The p38 MAPK activation is necessary for cancer cell death initiated by various anticancer agents [38,39,40]. Consistent with these findings, the present study observed that activation of p38 MAPK, but not ERK and JNK, was involved in paroxetine-induced cell death and that p38 MAPK activation was abolished by a ROS scavenger. ROS generation and p38 MAPK activation are essential to the death of MCF-7 cells in response to paroxetine treatment. However, there was no combination effect of NAC and SB203580 on cell viability and ROS inhibition. NAC showed stronger inhibitory effect on cell death than SB203580, but vice versa in ROS generation and MMP decrease. These results suggest that different signaling pathways might be present between cell viability and ROS generation/MMP changes.

### 3.2. Potential Role of Paroxetine in Breast Cancer

Although several chemotherapeutic drugs have been approved for treatment of breast cancer, these drugs have toxic side effects, and neoplasms can acquire resistance to these drugs [41]. To obtain better clinical results in the treatment of breast cancer, it is necessary to research secondary therapeutic agents that have less toxicity. Diminishing side effects and maximizing drug efficacy are major goals in cancer treatment. Paroxetine could be that chemotherapeutic agent because paroxetine has less toxicity in normal breast cells and exerts anticancer activity against MCF-7 and MDA-MB-231 cells. In addition, combination of paroxetine and chemotherapeutic drugs showed a synergistic effect on MCF-7 and MDA-MB-231cells.

Paroxetine is an SSRI that is as effective as other SSRIs and antidepressants in treating major depressive disorders [42,43]. However, researchers’ interest in paroxetine has decreased because of numerous side and adverse drug effects [43]. Paroxetine strongly binds to muscarinic receptors, noradrenaline transporter receptors, and serotonin receptors compared to other SSRIs [44]. However, the drug’s binding properties could be suitable for other purposes. Muscarinic receptors are involved in cancer cell proliferation, and acetylcholine (ACh) acts as a growth factor to stimulate cell growth [45]. When paroxetine binds to a muscarinic receptor, ACh activity is likely to be reduced. Paroxetine, among other antidepressants, was known as the strongest inhibitor of cytochrome P450 (CYP) 2D6 enzymes, by pharmacokinetic interactions, so tamoxifen’s anticancer effects are blunted and the risk for breast cancer recurrence is increased [43]. However, re-analysis has been demonstrated that paroxetine does not increase risk for cancer recurrence compared to other antidepressants [46,47,48].

The concentrations of 10 and 30 μM paroxetine induced cell death of MCF-7 and MDA-MB-231 breast cancer cells in this study. However, these concentrations are higher than that of therapeutic concentration of paroxetine. Therapeutic reference range for plasma concentrations of paroxetine in patients with major depressive disorders is 20–60 ng/mL when 10–40 mg/d of paroxetine for 6 weeks were administered as single daily dose. The dose can be increased in some patients not responding to the treatment dose [49]. The therapeutic concentration of paroxetine would not be expected to induce cell death much in vivo. However, in vivo, antidepressants should be used over a long period of time to get their effectiveness. Long-term exposure with low dose of paroxetine could induce cell death in vivo. Here, we cannot clearly compare the effect of paroxetine on cell death in vitro and in vivo because of the difference between short-term exposure with high dose and long-term exposure with low dose. Further study is needed to identify the effect of paroxetine treated for a long period of time with therapeutic concentration on breast cancer cells.

There are no completely safe drugs in the anticancer treatment, and the drugs which have the best effect on cancers could show lethal side effects. Although the efficacy of paroxetine on the treatment of depression in people with cancer is still unclear, antidepressants are a relatively safe and effective for severe depression in cancer patients. In addition, the current study adds anticancer effect of paroxetine on breast cancer cells to existing paroxetine database. Our findings indicate that paroxetine could be a potential anticancer agent rather than treatment for antidepressants in cancer patients with depression, and that the proper use of paroxetine could be helpful for the treatment of breast cancer regardless of depression. However, more research is needed to confirm the efficacy of paroxetine in breast cancer, and to find the right antidepressants for depression in people with cancer or only depression and cancer.

## 4. Materials and Methods

### 4.1. Chemicals and Stock Solutions

Unless otherwise stated, all chemicals were purchased from Sigma (St Louis, MO, USA). Stock solutions of amitriptyline (100 mM), bupropion (100 mM), citalopram (10 mM), EGTA (50 mM), fluoxetine (10 mM), and *N*-acety-l-cysteine (NAC, 300 mM) were prepared in distilled water (DW). 5-fluorouracil (100 μg/mL), doxorubicin (2 mg/mL), paroxetine (20 mM), PD98059 (20 mM), SB203580 (20 mM), SP600125 (20 mM), taxol (1 mg/mL), tianeptine (100 mM), and Z-VAD-FMK (10 mg/mL, InvivoGen, San Diego, CA, USA) were dissolved in dimethyl sulfoxide (DMSO). The solutions were then diluted in culture medium to the working concentration. Where DMSO was used as a solvent, solution containing equivalent concentration of DMSO was used as a control. Final DMSO concentrations for chemicals indicated above were ~0.1% (*v*/*v*).

Dulbecco’s modified Eagle’s medium (DMEM/F12), DMEM, fetal bovine serum (FBS), and horse serum were purchased from Gibco-BRL (Gaithersburg, MD, USA). RPMI-1640 was purchased from Lonza (Walkersville, MD, USA). Total glutathione assay kit was purchased from Assay Designs (Ann Arbor, MI, USA). FLICA apoptosis detection kit was purchased from Immunochemistry Technologies (Bloomington, MN, USA). MitoSox was purchased from Invitrogen (Carlsbad, CA, USA). JC-1 mitochondrial membrane potential detection kit was purchased from Biotium Inc (Hayward, CA, USA).

### 4.2. Cell Culture

Human breast cancer cell line MCF-7 were obtained from the Korean Cell Line Bank (Seoul, Korea), and corresponding normal breast epithelial cell line MCF-10A cells were kindly given by professor Aeri Mun (Duksung Women’s University, Seoul, Korea). MCF-7 cells were cultured in RPMI-1640 supplemented with 5% heat-inactivated FBS (Gibco) and antibiotics (100 U/mL penicillin and 100 µg/mL streptomycin: Pen-Strep, Gibco). MCF-10A cells were cultured in DMEM/F12 (Gibco) supplemented with 5% heat-inactivated horse serum, 0.5 μg/mL hydrocortisone, 10 μg/mL insulin, 20 ng/mL EGF, 2 mM L-glutamine, and antibiotics (Pen-Strep and 0.5 μg/mL amphotericin B). The cells were incubated at 37 °C in a 95% air-5% CO_2_ gas mixture. The medium was replaced every two days.

### 4.3. Cell Viability Assay

Cell viability was determined colorimetrically using 3-(4,5-dimethylthiazole-2-yl)-2,5-diphenyl tetrazolium bromide (MTT) reagent (5 mg/mL in PBS). MCF-7 and MCF-10A cells in the exponential phase were seeded (4 × 10^4^ cells/mL) in a 96-well plate (100 μL/ well), and the cells were cultured for 48 h before treatment with antidepressants indicated. After antidepressant treatment (12 to 72 h), MTT solution (10 μL) was added to each well and incubated for 3 h. The supernatants were aspirated, and the formazan crystals in each well were dissolved in DMSO (100 μL) for 30 min at room temperature. The 96-well plates were read at 570 nm with a microplate reader (Molecular Devices Inc., Sunnyvale, CA, USA).

### 4.4. Flow Cytometry Analysis

Apoptosis was determined by cell cycle analysis. For analysis of the cell cycle profile, MCF-7 cells (5 × 10^5^ cells/60-mm dish) were cultured for 48 h in RPMI medium, and the cells were harvested after 12 or 24 h treatment with paroxetine by trypsinization and by centrifugation at 168× *g* (1000 rpm) for 5 min. The cells were then fixed in 70% (*v*/*v*) ethanol and washed with ice-cold PBS. Whole cells were incubated with propidium iodide (PI) staining solution (10 mM Tris (pH 8.0), 1 mM NaCl, 0.1% NP40, 0.5 μg/mL RNase, and 0.05 mg/mL PI) in the dark for 30 min at 37 °C. Cellular DNA content and apoptotic cells based on the PI signal and sub-G1 peak were measured using a FACSCalibur™ system (Becton Dickinson, San Jose, CA, USA) collecting 10,000 events per sample and analyzed using CellQuest Pro™ software (Becton Dickinson). For analysis of apoptotic and necrotic cell death, the cells were labeled using a FITC Annexin V Apoptosis Detection Kit I (BD Pharmingen™, San Diego, CA, USA). FITC-conjugated annexin V (5 µL) and PI (5 µL) were added in 100 µL of breast cancer cells (1 × 10^5^), and then incubated for 15 min at room temperature in the dark. Following incubation, the cells were analyzed using a FACSCalibur™ system (Becton Dickinson).

### 4.5. Measurement of Caspase Activity

A fluorochrome inhibitor of caspase (FLICA) apoptosis detection kit was used to detect active caspases (Immunochemistry Technologies, Bloomington, MN, USA). Green fluorescent–labeled carboxyfluorescein derivative of valylalanylaspartic acid fluoromethyl ketone (FAM-VAD-FMK) is a potent inhibitor of caspase activity. Briefly, MCF-7 cells were incubated with 30 μM paroxetine for 12 h at 37 °C. Diluted FAM-VAD-FMK (1:30, 10 µL) was added to the 300 µL aliquot of each sample that was grown at a concentration of 1 × 10^6^ cells/mL. The cells were then labeled with FAM-VAD-FMK for 60 min at 37 °C. Cells were washed three times with 1× PBS. Caspase activity was detected using a band pass filter with excitation at 488 nm and emission at 518 nm.

### 4.6. Measurement of Intracellular Ca^2+^ Concentration

The intracellular Ca^2+^ was measured by a confocal laser scanning microscope equipped with a fluorescence system (IX70 Fluoview, Olympus, Tokyo, Japan). MCF-7 cells cultured on a corverglass bottomed dish (SPL, Pocheon, Korea) were incubated with 5 μM fluo-3AM in serum free RPMI media or normal physiological solution for 30 min and washed three times with 1× PBS. Each fluorescent image was scanned every 5 s at 488 nm on an excitation argon laser and 530 nm long pass emission filters. All scanned images were processed to analyze changes in intracellular Ca^2+^ concentration [Ca^2+^]_i_ at a single-cell level. In each cell studied, the changes in [Ca^2+^]_i_ were calculated as fluorescence intensity (F) divided by the basal fluorescence intensity before treatment (F0) in order to control for variations in basal fluorescence (F/F0). Net changes in F were represented as (Fmax−F0)/F0. Fmax is the maximum level of fluorescence intensity which occurred after the addition of paroxetine. The changes in [Ca^2+^]_i_ were measured for 8 min after treatment with paroxetine because the change in [Ca^2+^]_i_ is immediate reaction in response to chemicals.

### 4.7. Measurement of Intracellular Reactive Oxygen Species (ROS) Levels

Intracellular ROS generation was measured using dichlorodihydrofluorescein (H_2_DCFDA, Calbiochem, San Diego, CA, USA). MCF-7 cells were plated in a six-well plate and incubated with 5 μM H_2_DCFDA at 37 °C for 30 min in the dark. After incubation, the cells were washed three times with PBS and resuspended in PBS. The cell fluorescence was immediately analyzed using IX70 Fluoview (Olympus) and a flow cytometer (Becton Dickinson). For the detection of green fluorescence (H_2_DCFDA), cells were illuminated with 488 and 518 nm laser lines. Fluorescent images were analyzed using the Fluoview software program (version 2.0, Olympus). The time of paroxetine treatment to the cells could be changed to induce the strongest ROS generation. The amount of ROS generation was measured at different time during 12 h treatment with paroxetine. 

### 4.8. Measurement of Glutathione (GSH) Concentration

The glutathione concentration of the MCF-7 cells was measured by a total glutathione assay kit using a dithionitrobenzoic acid-glutathione disulfide (DTNB-GSSG) reductase recycling assay system (Assay Designs, Ann Arbor, MI, USA). MCF-7 cells were incubated with paroxetine for 12 h at 37 °C. The suspension of homogenized cells was mixed with GSH reductase in a microplate. The GSH concentration was measured ten times at 1 min interval at a wavelength of 405 nm using a microplate reader (Molecular Devices Inc.). The reduced GSH concentration was obtained by subtraction of the oxidized GSH (GSSG) concentration, which was recorded from samples treated with 4-vinylpyridine from the total GSH concentration. The following equation was used: reduced GSH = total GSH−GSSG.

### 4.9. Measurement of Mitochondrial ROS

Mitochondrial ROS levels were quantified according to the manufacturer’s protocol. MCF-7 cells were plated on a coverglass bottomed dish (SPL) at 48 h prior to the experiments. H_2_DCFDA (Calbiochem) and MitoTracker CMXRos (Invitrogen) were used to monitor the colocalized mitocondrial ROS level with green and red fluorescence. The cells were treated with desired concentration of paroxetine for 3 h, incubated with 100 nM CMXRos and 5 μM H_2_DCFDA at 37 °C for 30 min, and then washed with PBS. To detect more specifically mitochondrial ROS, MitoSOX red reagent (Invitrogen) was used. MCF-7 cells were treated with desired concentrations of paroxetine for 6 h. The cells were incubated with 5 µM MitoSOX for 10 min, washed with PBS, and collected. Fluorescence intensity was measured using IX70 Fluoview (Olympus).

### 4.10. Measurement of Mitochondrial Membrane Potential (MMP) and Plasma Membrane Potential

MMP changes were determined by JC-1 mitochondrial membrane potential detection kit (Biotium Inc.) according to the manufacturer’s protocol. Briefly, MCF-7 cells (5 × 10^5^/60-mm dish) were harvested after treatments with 10 or 30 μM paroxetine for 12 h and were stained with 1X JC-1 reagent at 37 °C for 15 min and resuspended with 1X PBS. Changes in MMP were measured at the single cell level using the FACSCalibur™ flow cytometer under the following conditions: FL1, 511 volts; FL2, 389 volts; FL1–10.5% FL2; FL2–25.9% FL1; 488 nm argon excitation lasers and 585 nm band pass filter. Total 10,000 cells were acquired for analysis using CellQuest software. Of paroxetine-treated cells, some cells were used for fluorescence image analysis. In non-apoptotic cells, JC-1 enters into the negatively-charged mitochondria where it aggregates and turns red, but in cells undergoing apoptosis, JC-1 exists as monomers in the cytosol and turns green because MMP has collapsed. Fluorescence intensity in a single cell was measured to compare MMP before and after paroxetine treatment. Mitochondrial membrane depolarization was usually monitored by a decrease in the fluorescence intensity ratio (red/green).

The change in plasma membrane potential (PMP) was measured with DiO PMP dye (Invitrogen) using the IX70 Fluoview (Olympus). MCF-7 cells cultured on corverglass bottomed dish (SPL) were incubated with 5 μM DiO dye in serum free RPMI-1640 media for 10 min, and washed three times with RPMI-1640 media. Each fluorescent image was scanned every 5 s at 488 nm on an excitation argon laser and 530 nm long pass emission filters. All scanned images were processed to analyze changes in PMP at a single-cell level.

### 4.11. Western blot Analysis

MCF-7 cells (5 × 10^5^/60-mm dish) were treated with the desired concentrations of paroxetine for the indicated time periods. The cells were vortexed in a protein extraction solution (PRO-PREPTM, iNtRON Biotechnology Inc, Seongnam, Korea) containing 50 mM Tris-Cl (pH 7.5), 150 mM NaCl, 1 mM dithiothreitol, 0.5% nonyl phenoxylpolyethoxylethanol, 1% Triton X-100, 1% deoxycholate, 0.1% sodium dodecyl sulfate (SDS), 1 mM EDTA, and 1× protease inhibitor cocktail (Roche Diagnostics, Indianapolis, IN, USA). The cell lysates were incubated for 30 min on ice with intermittent vortexing and were clarified by centrifugation at 16,609× *g* (13,000 rpm, Hanil, Incheon, Korea) at 4 °C for 20 min. After centrifugation, the supernatant was separated and stored at −70 °C until use.

Mitochondrial and cytosolic fractions were isolated using mitochondria/cytosol fractionation kit (Biovision, Mountain View, CA, USA) according to the manufacturer’s protocol. MCF-7 cells treated with paroxetine collected by centrifugation at 598× *g* (2500 rpm) and 4 °C for 5 min. The cells were then washed twice with ice-cold PBS (pH 7.4), followed by centrifugation at 598× *g* for 5 min. The cell pellet was resuspended in 1 mL of 1× cytosolic extraction buffer containing 1 mM dithiothreitol (DTT) and protease inhibitors. After incubation on ice for 10 min, the cells were homogenized with a glass dounce homogenizer. The homogenates were subjected to centrifugation at 598× *g* and 4 °C for 10 min to remove intact cells, cell nuclei, and cell membrane. The resulting supernatant was then subjected to centrifugation at 9809× *g* (10,000 rpm) for 30 min to collect the mitochondrial and cytosolic fractions. The resulting supernatants were stored at −80 °C (cytosolic fraction), and the pellets were resuspended in 0.1 mL mitochondrial extraction buffer (mitochondrial fraction) until use.

Protein concentration was quantified in cell lysates using BCA protein assay kit (Thermo scientific, Rockford, IL, USA). Equal amounts of proteins were mixed with 5× sample buffer, separated with 8% or 10% SDS-polyacrylamide gel, and transferred to polyvinylidene difluoride (PVDF, Millipore, Billerica, MA, USA) membrane for 15 min using a semi-dry transfer (Bio-Rad, Hercules, CA, USA). Membranes were blocked with 5% (*w*/*v*) fat-free dry milk in TBST (20 mM Tris HCl (pH 8), 137 mM NaCl, and 0.2% Tween-20) at room temperature for 60 min and then incubated with desired primary antibodies for apoptosis-related proteins; Bcl-2, Bcl-xL, Bax, caspase-8, caspase-9, cytochrome C, VDAC (Cell Signaling, Danvers, MA, USA), and PARP (Cell signaling) and cleaved PARP, mitogen activated protein (MAP) kinases-related proteins; p-ERK1/2, ERK1/2, p-JNK, JNK, p-p38, and p38, and α-tubulin at 4 °C overnight. The primary antibody incubation was followed by incubation with a secondary horseradish peroxidase (HRP)-conjugated anti-rabbit or anti-mouse antibody at 1:10,000. Immuno-positive bands were visualized by enhanced chemiluminescence (ECL Plus kit, ELPIS, Taejon, Korea) following the manufacturer’s instructions. Relative protein level was calculated using α-tubulin as a loading control.

### 4.12. Statistical Analysis

Data are represented as mean ± SD. Significant differences between groups were analyzed by using one-way ANOVA with post-hoc comparisons using Tukey’s test (SPSS v18, Chicago, IL, USA). Differences were considered significant at *p* < 0.05.

## 5. Conclusions

In conclusion, paroxetine, among antidepressants tested in this study, induced apoptosis of human breast cancer MCF-7 cells through extracellular Ca^2+^- and p38 MAPK-dependent ROS generation highly compared to normal cells. In addition, paroxetine gives synergistic effect on cell death by chemotherapeutic drugs. These results suggest that paroxetine may serve as an anticancer adjuvant to current cancer therapies for breast cancer patients with or without depression. These findings give new insight into the role of paroxetine in breast cancer.
